# Prevalence of hypovitaminosis D among children and adolescents of Kabul: a descriptive cross-sectional study

**DOI:** 10.1186/s12887-023-03861-1

**Published:** 2023-02-01

**Authors:** Mohammad Sharif Sediqi, Abdul Rasheed Mansoor, Mohmand Mangal

**Affiliations:** 1 Department of Pediatrics, Kabul University of Medical Sciences (KUMS), Kabul, Afghanistan; 2 Ariana Medical Complex (AMC), Pediatrics Department, Kabul, Afghanistan

**Keywords:** Vitamin D deficiency, Kabul, Prevalence, Ariana Medical Complex, Kabul University of Medical Sciences, Children

## Abstract

**Background:**

Vitamin D is one of the most important fat-soluble vitamins necessary for normal growth and development of the human body. According to a study done in Kabul shows that economic, racial, and social concerns are thought to be the main impediments to receiving appropriate amounts of this vitamin through dietary sources in countries like Afghanistan. Hypovitaminosis D, on the other hand, is now recognized as a pandemic in both industrialized and developing countries.

**Methods:**

To find out how common hypovitaminosis D is in children aged one month to eighteen years in afghan children Kabul, Afghanistan. Vitamin D deficiency and insufficiency are defined as serum levels of less than 20 ng/mL and 20 to 30 ng/mL, respectively. Children aged between 1 month to 18 years attending our hospital, AMC (Ariana Medical Complex) for health examination were checked for their 25-hydroxyvitamin D [25(OH)D]. Age, gender and address were recorded. 25(OH)D were determined using immunoassay auto analyzers. According to their serum 25(OH)D, the 25(OH)D were categorized into five categories: sufficiency: ≥ 30-100 ng/mL; insufficiency: ≥ 20-29 ng/mL; deficiency: < 20 ng/mL; severe deficiency: < 10 ng/mL; and intoxication: > 150 ng/mL. Participants who were intoxicated with vitamin D were excluded from the study.

**Results:**

A total of 4008 children aged 1 month to 18 years participated in this cross-sectional study. Hypovitaminosis D was found to be prevalent in 62.5 percent of the population. When compared to boys, female children were 1.2 times more likely to be vitamin D deficient. When compared to children of illiterate women, the odds of hypovitaminosis D were 1.4, 1.9, and 5.8 times lower in children with mothers educated up to primary school, graduation, and post-graduate. The average vitamin D level was 23 ng/mL, with a median of 15 ng/mL and maximum and minimum values of 135 ng/mL and 3 ng/mL, respectively.

In all, 2500 (62.5%) of the children had low levels of vitamin D in their serum. Only 400 (16%) of the patients were sufficient, whereas 917 (36.7%) were severely deficient, 733 (29.3%) were deficient, and 450 (18%) were insufficient. With a female to male ratio of 1.2:1, the majority of those, 1335 (53.4%), were females and 1165 (46.6%) were males. Patients were 8.14 years old on average, with a median age of 7 years. The majority of the patients, 2152 (86.1%), were urban, while 348 (13.9%) were rural.

**Conclusion:**

The prevalence of hypovitaminosis D was very high in Afghan children. Female sex, higher socio economic status, higher educational status of the mother and living at urban areas were the factors with strong positive association with hypovitaminosis D.

## Introduction

Vitamin D is one of the most important fat-soluble vitamins necessary for normal growth and development of the human body. Sunlight is the principal source of vitamin D in the human body. UVB rays from the sun interact with 7-dehydrocholesterol in the skin to form this substance. According to a study done in Kabul shows that Economic, racial, and social concerns are thought to be the main impediments to receiving appropriate amounts of this vitamin through dietary sources in countries like Afghanistan. Hypovitaminosis D, on the other hand, is now recognized as a pandemic in both industrialized and developing countries [1].

According to several studies, vitamin D insufficiency is frequent among people in the United States, and it is more prevalent in the winter and spring than in the fall and summer. In a survey of Turkish women, it was discovered that those who wore religious clothing had vitamin D insufficiency [2].

In healthy children in Tehran, vitamin D insufficiency was found to be widespread, with females having a greater frequency than boys [3, 4]. Hypovitaminosis D is also common in Pakistan, according to studies undertaken in various sections of the country [5, 6]. Hypovitaminosis D is also prevalent in India's youth, according to studies from various sections of the nation [7].

Inadequate 25(OH)D are pretty frequent all around the world. However, information on vitamin D status in Afghan children are insufficient due to a paucity of research on the topic. As a result, the goal of this research was to find out how common vitamin D insufficiency is among children who appear to be healthy and attend a tertiary care facility in Kabul.

## Methodology

This research was carried out in pediatrics department of Ariana Medical Complex Hospital located in Kabul, Afghanistan over a period of one year between years 2020 to 2021. The Ethical Committees of the AMC and Kabul University of Medical Sciences (KUMS) gave their approval to the study. All children including boys and girls aged 1 month to 18 years old who came to the Hospital during this period were included in the research. 25(OH)D were determined using immunoassay auto analyzers. According to their serum 25(OH)D, the 25(OH)D were categorized into five categories: sufficiency: ≥ 30-100 ng/mL; insufficiency: ≥ 20-29 ng/mL; deficiency: < 20 ng/mL; severe deficiency: < 10 ng/mL; and intoxication: > 150 ng/mL. Participants who were intoxicated with vitamin D were excluded from the study. A total of 4008 children aged 1 month to 18 years participated in this cross-sectional study over this period. Due to vitamin D intoxication 25(OH)D level > 150 ng/mL, eight of the patients were excluded from the research, leaving a sample size of 4000 for the final analysis. The parents of the children gave their written consent after receiving all of the relevant information regarding the study and various clinical and demographic aspects of children, like age, gender, economic position, mother's educational background, and current residence, were observed in order to uncover factors connected to vitamin D deficiency.

Each participant's 25(OH)D level was determined by taking 5 mL of serum and putting it in a Tube at the time of visit. The serum 25(OH)D was measured using a chemiluminescence immunoassay(Biomerieux, Mini VIDAS, Paris, France, accurate and standard) after the sample tube was centrifuged. Based on 25(OH) D concentrations in the serum, the individuals were classified into five groups: 1) Sufficiency: ≥ 30–100 ng/mL; 2) Insufficiency: ≥ 20–29 ng/mL; 3) Deficiency: < 20 ng/mL; 4) Severe Deficiency: < 10 ng/mL; 5) Intoxication: > 150 ng/mL. Version 28 of (IBM SPSS Statistics, California, USA) free trial was used to conduct analysis.

## Results

There were 4008 children approached in all and eight of the subjects were eliminated from the study due to vitamin D toxicity [25(OH)D level > 150 ng/mL], thus, the study had a 0.2% non-participation rate. The study involved a total of 4000 children. 25(OH)D ranged from 3.4 ng/mL to 135 ng/mL, with a mean of 23 ng/mL and a median of 15 ng/mL, respectively.

In fact, 2500 (62.5%) of the children had insufficient amounts of vitamin D in their serum (Table 1). Only 400 (16%) of the subjects had sufficiency, while 917 (36.7%) had severe deficiency, 733 of the children have vitamin D deficiency ( 29.3%), Vitamin D insufficiency was found in 450 individuals (18 percent) (Table 2). With a 1.2:1 female-to-male ratio, the majority of those 1335 (53.4%), were females and 1165 (46.6%) were males (Table 3). of the total number of people that took part in the research, 682 (27.3%) were less than five years, 998(39.9%) were 5 to 9 years, 460(18.4%) between the ages of 10 and 14 and the remained 360 (14.4%) were over 15 years of age (Table 4). The patients were on average 8.14 years old, with a median age of 7 years. The majority of the patients, 2152 (86.1%), were urban, while 348 (13.9%) were rural (Tables 5 & 6).

**Table 1:** Prevalence of Vitamin D insufficiency

Vitamin D Level	Frequency	Percentage
Low levels of Vitamin D	2500	62.5
Total	4000	100

**Table 2:** Distribution of Vitamin D levels

Vitamin D Level	Frequency	Percentage
Deficiency	733	29.3
Severe Deficiency	917	36.7
Sufficient	400	16
Insufficient	450	18
Total	2500	100

**Table 3:** Distribution by gender

Gender	Number of children	Percentage
Male	1335	46.6
Female	1165	53.4
Total	2500	100

**Table 4:** Distribution of children studied by age

Age	Number of children	Percentage
Below 5 years	682	27.3
5–9 years	998	39.9
10–14 years	460	18.4
15 and above	360	14.4
Total	2500	100

**Table 5:** Distribution of vitamin D level based on address

Address	Frequency	Percentage
Rural	348	13.9
Urban	2152	86.1
Total	2500	100

**Table 6:** Hypovitaminosis D with a variety of sociodemographic factors (*N* = 2500)

Parameter	Sufficient (*N* = 400)(16%)	Insufficient (*N* = 450)(18%)	Deficient (*N* = 733)(29.32%)	Severe deficient (*N* = 917)(36.7%)
**Age**				
Below 5 years	133(33.3%)	168(37.3%)	148(23.1%)	314(34.3%)
5–9 years	146(36.6%)	228(50.7%)	390(61.0%)	522(56.9%)
10–14 years	67(16.6%)	47(10.4%)	80(12.6%)	47(5.1%)
15 and above	54(13.3%)	7(1.5%)	20(3.1%)	34(3.6%)
**Sex**				
Male	66(16.6%)	161(35.8%)	121(18.9%)	228(24.8%)
Female	334(83.3%)	289(64.1%)	517(81.0%)	689(75.1%)
**Economic status**				
Lower	100(2%)	161(35.8%)	221(34.7%)	248(27%)
Upper lower	66(16.6%)	40(8.9%)	148(23.1%)	167(18.4%)
Lower middle	53(13.3%)	148(32.8%)	101(15.7%)	201(21.9%)
Upper middle	147(36.6%)	54(11.9%)	134(21.0%)	234(25.4%)
Upper	33(8.3%)	47(10.4%)	34(5.2%)	67(7.3%)
**Education of mother**				
Illiterate	66(16.6%)	114(25.3%)	154(24.2%)	221(24.0%)
Up to primary	100(2%)	134(29.8%)	67(10.5%)	201(21.9%)
Primary to + 2	133(33.3%)	87(19.4%)	134(21%)	67(7.3%)
Graduate	47(11.6%)	80(17.9%)	215(33.6%)	314(34.3%)
Postgraduate	53(13.3%)	34(7.4%)	67(10.5%)	114(12.4%)
**Address**				
Rural	313(78.3%)	369(82%)	463(72.6%)	529(57.6%)
Urban	87(21.6%)	81(17.9%)	175(27.3%)	388(42.3%)

Based on modified Kuppuswamy’s socioeconomic status scale [8], 315 (12.6%) of the children belonged to the high socioeconomic class, 733 (29.3%) to the upper medium, 590 (23.6%) to the lower middle, 683 (27.3%) to the upper lower, and the remainder 282 (11.3%) to the lower socioeconomic class (Tables 6 & 7). 382 (15.3%) of mothers were illiterate, 463(18.5%) having finished elementary school, 698(27.9%) attended school from six to twelve years, 717(28.7%) were graduated and 240(9.6%) had post graduate degrees’ (Table 8). 315 (12.6%) of children were underweight, 1709 (68.3%) nutrition was normal and the remaining 95 (3.8%) had weight over normal range.

**Table 7:** Socioeconomic wise distribution of families

**Economic status**	Frequency	Percentage
Upper	315	(12.6%)
Upper middle	733	(29.3%)
Lower middle	590	(23.6%)
Upper lower	682	(27.3%)
Lower	283	(11.3%)

**Table 8:** Education of mothers

Education of mother	Frequency	Percentage
Illiterate	382	(15.3%)
Up to primary	463	(18.5%)
Primary to + 2	697	(27.9%)
Graduate	718	(28.7%)
Postgraduate	240	(9.6%)

## Discussion

According to the study, vitamin D deficiency is common (62.5 percent) among Afghan children, with the criteria of vitamin d deficiency being [25(OH) D < 20 ng/mL]. Based on the results, the vitamin D level of children in Kabul city is inadequate. Vitamin D insufficiency is a concern for Afghan children. Vitamin D supplementation during pregnancy raises maternal and cord serum 25(OH) D level [9]. another study found that Bangladesh infants start their lives with low 25(OH)D due to low maternal prenatal 25(OH) D, which was seen in both urban and rural Bangladeshi women of reproductive age [10]. In an Indian study of 35 three-month-old breastfed neonates, 51 percent had 25(OH) D levels of 15 ng/mL, with a mean of 20 ng/mL [11]. In Pakistan, 38 breastfed infants aged 6 months had a mean 25(OH) D level of 10 ng/mL, whereas 71% of infants aged less than three months (12/17) had 25(OH) D levels less than 16 ng/mL [12]. Furthermore, another study from the Middle East found that 82 percent of 78 breastfed term children, aged 1 to 4 months, 25(OH)D levels were 10.01 ng/mL in children born to moms who did not drink enough milk and had a habit of covering their skin fully when outside, with a median 25(OH)D of 5 ng/Ml [13]. When our findings are compared to those of an American research, we discover that our children and adolescents have a significantly higher prevalence of vitamin D insufficiency than children and adolescents in the United States. The American Academy of Pediatrics announced a new guideline in November 2008, recommending that all children eat 400 IU of vitamin D each day from birth until puberty [15]. As a result, we support the plan of vitamin D supplementation for Afghan children should be adopted, as vitamin D insufficiency in Afghan children is more severe than other ethnic groups. Our current research has some limitations. The participants were not drawn from Afghanistan's entire pediatric population. There were no data collected on probable vitamin D status relevant factors such as supplement consumption, lifestyle, or food habits. A second study based on people recruited from the general community would be conducted, and the possible vitamin D-related factors must be investigated. Hypovitaminosis D is becoming more widely recognized as a pandemic affecting, both developed and developing countries.

Vitamin D is mostly obtained through sunlight. Only a few foods naturally contain vitamin D, and fortified foods are insufficient to satisfy vitamin D requirements. Hypovitaminosis D [25(OH)D level < 30 ng/mL] was frequent in many regions of the world, according to the International Osteoporosis Foundation (IOF), whereas severe vitamin D insufficiency [25(OH)D level < 10 ng/mL] was most common in South Asia and the Middle East [14].

This is the primary study that evaluates 25(OH)D in Afghan children. According to our data, over three-quarters of Afghan youngsters in Kabul province suffer from hypovitaminosis D to varying degrees. There is a link between serum 25(OH)D concentrations and gender, according to our findings. Female children were found to have a higher rate of severe vitamin D insufficiency than boys. Many investigations in neighboring countries yielded similar results [2, 4].

Reduced sun exposure, religious clothing (which covers the entire body), sun protection, and more time spent indoors are all possible reasons of severe vitamin D insufficiency in female teens. So, more research is needed to know what causes Hypovitaminosis D in Afghan female children. The impact of sunshine on 25(OH)D in humans has been thoroughly studied, proving the importance of UV-induced vitamin D synthesis. The avoidance of sunshine for fear of skin darkening and the deliberate covering of the complete body when going outside, especially in ladies, were significant causes.

Males avoid sun exposure as well, due to a misconception of sunlight's harmful effects and a lack of awareness of where Vitamin D comes from. Our findings matched those of previous investigations. As well as, one of the study's shortcomings was that the 25(OH)D level in the serum was only assessed once, which may not adequately reflect vitamin D state throughout the year; to examine seasonal variance, at least two samples should have to be taken in different months of a year. This article adds to the data revealing a significant prevalence of vitamin D deficiency among Afghan children, and it urges for more investigations. Health care providers should urge youngsters to live a healthy lifestyle that includes plenty of outdoor activities for maximum sun exposure and a vitamin D-rich diet. National health policies that encourage vitamin D level screening, public education about the significance of vitamin D, prevention through fortified food, and treatment with vitamin D supplements are all long-term measures to address this issue.

## Conclusion

Vitamin D deficiency is quite common in the pediatric population, especially among adolescents and female children. It's more common among the upper socioeconomic levels families, which is likely due to decreased exposure to sunshine. Based on this high prevalence of vitamin D deficiency and despite the fact that vitamin D is a sunlight vitamin, it is critical to provide appropriate vitamin D supplementation to the Afghan population. Vitamin D deficiency and insufficiency are particularly common among children in Kabul, which is concerning. More study is needed to explore among the national representative samples. Furthermore, because it is critical for a child's growth and development, the government must design a thorough action plan to prevent such deficiencies.

Vitamin D supplementation for Afghan children from infancy through adolescence is strongly recommended, and it should be implemented as soon as feasible by the government. In addition, raising awareness about vitamin D insufficiency and fortification of foods such as milk, oil, yogurt, and cereal, as well as ensuring the quality of the fortification, is critical for the prevention of vitamin D deficiency and its consequences in Afghanistan. The limited sample size of this study is a drawback; further research in a wider population is needed.

## Data Availability

The datasets generated and analysed during the current study available from corresponding author on reasonable request.

## References

[1] Azizi S, Tariq TM (2019). Vitamin D deficiency among Afghan adolescents in Kabul. J College Physicians Surg Pakistan.

[2] Hatun S, Islam Ö, Cizmecioglu F, Kara B, Babaoglu K, Berk F (2005). Subclinical vitamin D deficiency is increased in adolescent girls who wear concealing clothing. J Nutr.

[3] Tabrizi R, Moosazadeh M, Akbari M, Dabbaghmanesh MH, Mohamadkhani M, Asemi Z (2018). High prevalence of vitamin D deficiency among Iranian population: a systematic review and meta-analysis. Iran J Med Sci.

[4] Rabbani A, Alavian SM, Motlagh ME, Ashtiani MT, Ardalan G, Salavati A (2009). Vitamin D Insufficiency among children and children living in Tehran. Iran J Trop Pediatr.

[5] Mahmood K, Akhtar ST, Talib A, Haider I (2009). Vitamin-D status in a population of healthy adults in Pakistan. Pak J Med Sci.

[6] Iqbal R, Khan A (2010). Possible causes of vitamin D deficiency (VDD) in population residing in Pakistan. J Pak Med Assoc.

[7] Soliman A, Sanctis V, Elalaily R, Bedair S, Kassem I (2014). Vitamin D deficiency in children. Indian J Endocrinol Metab.

[8] Al-Daghri NM (2018). Vitamin D in Saudi Arabia: prevalence, distribution and disease associations. J Steroid Biochem Mol Biol.

[9] Islam MZ, Lamberg-Allardt C, Karkkainen M, Outila T, Salamatullah Q, Hamim AA (2002). Vitamin D Deficiency: a concern in premenopausal Bangladeshi women of two socio-economic groups in rural and urban region. Eur J Clin Nutr.

[10] Bhalala U, Desai M, Parekh P, Mokal R, Chheda B (2007). Subclinical hypovitaminosis D among exclusively breastfed young infants. Indian Pediatr.

[11] Atiq M, Suria A, Nizami SQ, Ahmed I (1998). Maternal vitamin D Deficiency in Pakistan. Acta Obstet Gynecol Scand.

[12] Dawodu A, Agarwal M, Hossain M, Kochiyil J, Zayed R (2003). Hypovitaminosis D and vitamin D deficiency in exclusively breast-feeding infants and their mothers in summer: a justification for Vitamin D supplementation of breast-feeding infants. J Pediatr.

[13] Wagner CL, Greer FR (2008). Prevention of rickets and vitamin D deficiency in infants, children, and adolescents. Pediatrics.

[14] Mithal A, Wahl DA, Bonjour JP, Burckhardt P, Dawson-Hughes B, Eisman JA (2009). IOF committee of scientific advisors (CSA) nutrition working group Global vitamin D status and determinants of hypovitaminosis D. Osteoporosis Int.

[15] Casey C, Slawson DC, Neal LR. Vitamin D supplementation in infants, children, and adolescents. American family physician. 2010;81(6):745-8. 20229973

